# Interference Effects Redress over Power-Efficient Wireless-Friendly Mesh Networks for Ubiquitous Sensor Communications across Smart Cities

**DOI:** 10.3390/s17071678

**Published:** 2017-07-21

**Authors:** Jose Santana, Domingo Marrero, Elsa Macías, Vicente Mena, Álvaro Suárez

**Affiliations:** 1 Grupo de Arquitectura y Concurrencia (GAC), Instituto Universitario de Ciencias y Tecnologías Cibernéticas (IUCTC), Universidad de Las Palmas de Gran Canaria, 35017 Las Palmas de Gran Canaria (ULPGC), Spain; elsa.macias@ulpgc.es (E.M.); vicenteefigenio.mena@ulpgc.es (V.M.); alvaro.suarez@ulpgc.es (Á.S.); 2 Departamento de Señales y Comunicaciones (DSC), Universidad de Las Palmas de Gran Canaria, 35017 Las Palmas de Gran Canaria (ULPGC), Spain; 3 Departamento de Ingeniería Telemática (DIT), Universidad de Las Palmas de Gran Canaria, 35017 Las Palmas de Gran Canaria (ULPGC), Spain

**Keywords:** ubiquitous sensing, wireless-friendly, interference, power saving, portable mesh network, smart city

## Abstract

Ubiquitous sensing allows smart cities to take control of many parameters (e.g., road traffic, air or noise pollution levels, etc.). An inexpensive Wireless Mesh Network can be used as an efficient way to transport sensed data. When that mesh is autonomously powered (e.g., solar powered), it constitutes an ideal portable network system which can be deployed when needed. Nevertheless, its power consumption must be restrained to extend its operational cycle and for preserving the environment. To this end, our strategy fosters wireless interface deactivation among nodes which do not participate in any route. As we show, this contributes to a significant power saving for the mesh. Furthermore, our strategy is wireless-friendly, meaning that it gives priority to deactivation of nodes receiving (and also causing) interferences from (to) the rest of the smart city. We also show that a routing protocol can adapt to this strategy in which certain nodes deactivate their own wireless interfaces.

## 1. Introduction

In smart cities the availability of ubiquitous sensor communications is essential, but the use of a flexible and portable network for transporting the sensed data is also key. A Wireless Mesh Network (WMN) [1,2] is a key technology in current wireless communications which fulfils this aim. It is arranged into a set of devices (user terminals or linking interfaces) whose interconnections are wireless and organized in a lattice structure. The International Electrical and Electronic Engineering (IEEE) association has developed different standards, especially IEEE 802.11 for Wireless Fidelity (WiFi) or IEEE 802.15 for Wireless Sensor Networks (WSNs), among others. They are generally deployed in an ad-hoc mode, depending on the requirements, and can be used as a means of transport for data from end users and specially for interconnection of subnetworks whose distance or difficult access require wireless connectivity. Often communication sources can be directly linked to mesh nodes, e.g., user smartphones cooperating in a shared task. In some solutions for smart cities there already exist different proposals for facilitating both mobile collaborative sensing and sensed data access, so the collected information can be processed and shared by all the users. As stated above, typical sensed data are generated by smartphone sensors (such as Global Positioning System (GPS) receivers, accelerometers, cameras and several others) but also from different sources like presence sensors (those which usually activate lighting). Whatever mission a WMN has (a traditional transport network, a network for sensing capable stations, or a sensor network), it entails multiple challenges such as routing, throughput, range of operation, power saving, adaptability, stability, and robustness, among others.

Some technologies at the link level have been proposed to implement WMNs [3] in different environments. The IEEE 802.11s [4] standard is normally used in wide area WMNs deployed in cities or places of similar area. WiFi routers and Access Points (APs) have been proposed to implement such WMNs [5]. We will call them hereafter Mesh Access Points (MAPs).

WMNs can be deployed in very large numbers in different domains [6], e.g., a smart city [7] in which the WMN is configured as a backbone that transports a variety of traffic including sensor data [8], and multimedia [9], among others, but there are still several challenges to convert them into a suitable infrastructure in some domain applications. Next we review some of them.

An efficient channel assignment in WMN [10] improves its throughput considerably. The optimal assignment is not efficient or impossible when there will be uncontrolled external aspects present around the WMN, e.g., the interferences caused by external APs and other wireless devices owned by a third party. Those devices can be commonly deployed statically or sometimes dynamically (e.g., massive usage of mobile telephones with tethering technology as in [11]).

Channel interferences or collisions in current cities [12], where dense WiFi networks are deployed, could be a hard problem for IEEE 802.11s- or WiFi-based WMNs. WiFi interferences penalize greatly data communication due to the fact wireless channels would be busy communicating signalling. The routing is a key parameter to obtain the connectivity among MAPs [13]. Optimized Link State Routing (OLSR) [14] is a routing protocol used in Mobile Ad Hoc NETworks (MANETs), but it also can be used in WMNs, as well as Hierarchical OLSR (HOLSR) [15]. When a strong pattern of collisions or interferences, signal or packet loss, connection drops, or even service disruptions [16] occur in a MAP, its suitability for relaying the packets of any route will be compromised. In that case, the routing protocol must rapidly set up a new route in order to maintain the connectivity of the network.

Energy saving in WMNs is another important challenge [17] as recognized in [18], but it is also a driving force behind sustainable solutions for smart cities [19,20,21,22,23,24]. We have presented various methods targeting power saving in different areas [25,26], considering setting up and taking down the wireless interfaces of the MAPs whenever possible. We showed that a significant amount of power could be saved by this. In this sense we are aligned with one of the main challenges of smart cities [27].

The challenges mentioned above are applicable to any WMN, but we will focus on WiFi-based WMNs viewed as a ubiquitous, portable, and scalable solution for any deployment requirement; targeting to allow end users to access sensing data, which are directly delivered by MAPs. Unlike major WSNs (based on Zigbee or IEEE 802.15.4), our network model overcomes their restrictions by extending their range of operation, rate and compatibility. Thus we will call it Portable Wireless-friendly Mesh Network for Sensors (PWMNS), which is conceived as a communication backbone, as well as a source and sink, for sensor data traffic in a smart city.

In [25,26] we reviewed power saving works from other authors that are complementary to our mechanism. In this paper we extend our previous works [25,26] considering a new strategy to set up and take down down the WiFi interfaces of MAPs. The strategy includes taking these WiFi interfaces down whenever a MAP detects a moderate level of interference from the other MAPs or external WiFi devices. We present new algorithms, protocol implementation and software architecture to accomplish this strategy. We also performed a simulation on new parameters measuring the obtained interference redress and power saving. Finally we obtained experimental results on a testbed after applying our strategy. In addition to obtain a power saving (like in [25,26]), we also redress the adverse effects of interferences inside the PWMNS and in external surrounding WiFi devices.

The rest of the paper is organized as follows: in Section 2 we present the background on related and previous works. Section 3 presents our system architecture for supporting sensor data traffic in a smart city. Section 4 sketches the mechanism and protocol for a MAP to controllably set its wireless interface up and take it down. Section 5 formalizes the interference redress and the power saving degrees achieved by the PWMNS; and simulation results shows that deactivation and activation of MAPs wireless interfaces can allow both redressing the effects of interferences, as well as power savings when they are not required in any route. Then, in Section 6, we present the experimental results over a test platform based on Raspberry Pi (RPi) [28]. Finally, conclusions are presented in Section 7.

## 2. Background and Related Work

Wireless networks are prone, by nature, to suffer from broken end to end communication service. This is due to the intrinsic nature of wireless communication channels that can randomly fade off (there is no way to exactly know in advance when it will faded off), support a high number of active services demanding a high bandwidth, or be subject to strong interferences from other channels or strong collision patterns. All those characteristics could reduce significantly the end to end throughput to the extent that affected MAPs (hereinafter interfered MAPs) links could be broken. A very strong interference of the above characteristics could produce an end to end communication service interruption. This provokes a headache for users consuming multimedia information (streaming), but could be also a big problem for traffic from critical sensors in a smart city (machine to machine or machine to human types of communication).

Solutions to the above problem are not simple to obtain, because it requires several levels of the network architecture to cooperate in the solution. That is, it is impossible to solve this problem only at the physical and link level. In general, cross layer techniques have to be applied [29]. We have worked in solutions to this problem for MANETs where over time the communication nodes can dynamically enter and leave the coverage area [30]. Briefly, in the intermediate nodes of the MANET we deployed the OLSR protocol. We configured the OLSR daemon (OLSRd) to calculate the optimal routes defined as the number of attempts by a node on average to successfully transmit a packet to a destination, instead of the number of hops. Using OLSRd, a node can detect changes in the connectivity to its neighbours by injecting and receiving HELLO messages periodically. OLSR is able to quickly reconfigure path breaks using an efficient flooding of control traffic. OLSR messages are not duplicated thanks to its multipoint relays. Finally OLSRd diffuses the topological information necessary to obtain optimal routes in terms of number of hops (this information is periodically renewed). An OLSRd plug-in allows the application level to inject user defined packets (type 200) in intermediate nodes using a default forwarding algorithm [31]. Spreading those messages the application can control the path breaks and interference by reconfiguration of the path. We call that application disruption control relay (dcr). In [32] it is shown that OLSR and HOLSR are efficient enough to reconfigure broken paths, in real time, for streaming services. In [33] it is shown that OLSR can be efficiently used to deploy WMNs over long distances supporting traffic of emergency critical applications in case of natural disasters. In [34] a routing algorithm based on OLSR for finding the best routes according the quality of WiFi links was presented. The simulation results are better than using the Expected Transmission Count metric, but they do not show that this will be optimal for dense networks. For Voice over Internet Protocol (VoIP) [35] efficient policies to assure the low delay data delivery and balancing the traffic among APs are required. We do not deal with these special requirements explicitly. An optimization of OLSR for heterogeneous WMN (APs have multiple wireless interfaces) is the Unified Routing Protocol (URP) that eliminates the redundant diffusion broadcast messages of OLSR [36]. Simulation results in [37] showed that URP is better than OLSR. We do not explicitly consider heterogeneous WMNs, but in case URP allows the usage of dcr it could be easy to contemplate this.

According to the above examples of application of OLSR to MANETs and WMNs for different types of traffic, we argue that it is efficient to set up or take down the wireless interfaces of the MAPs affected by external interferences, and rebuild broken routes efficiently. In the following sections we discuss the main idea about how to apply OLSR and dcr to our wireless network.

In any WiFi network, a MAP could degenerate into a bottleneck when it is affected by interferences, overlapped channels or collisions. To reduce this possibility, we propose to deactivate its wireless interface as long as the interference is present. Furthermore, we consider applying the mechanism presented in [25,26] as part of this proposal, so that the power savings are increased as well at the same time the negative effects of interferences are redressed.

## 3. System Architecture

Any MAP can be marked as (externally wireless) interfered by testing various measures like beacon detection (in the same or overlapped channels) from external sources, increased channel access time, delays, loss of connectivity, service disruptions, or any other criterion. As stated in Section 1, when a MAP is strongly interfered by external factors, the routing protocol is responsible for redressing their effects. On the other hand, in our background of a backbone delivering a low-requirement sensor-data traffic, it is common for many nodes not to be linked to any active route (hereinafter unused nodes). Our proposed approach intends to take the wireless interfaces of both unused and interfered nodes down. Thus we reach some power savings, as well as interference redress, when managing the routing protocol to prioritize the MAPs not (or only moderately) interfered. This allows for redressing external moderated interference which may ballast the PWMNS traffic in the long term, or furthermore, the reverse interference of PWMNS over external devices of the smart city. This latter redress justifies the term wireless friendly [38] in our PWMNS acronym.

**Figure 1:**
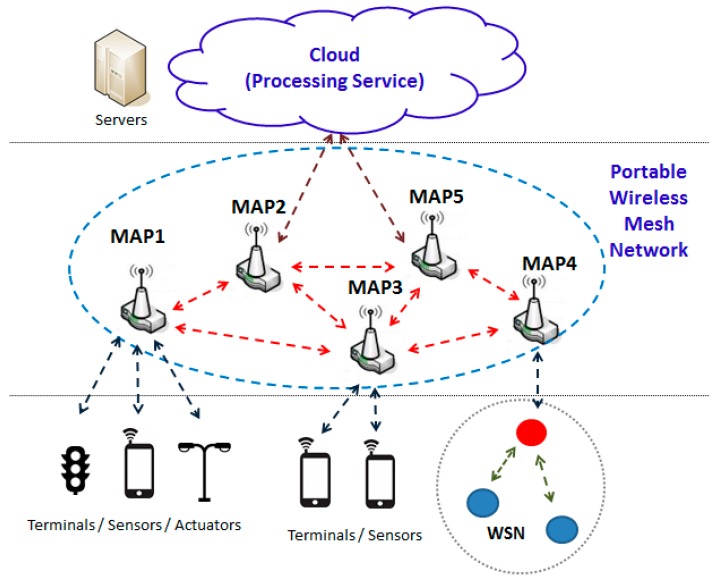
System architecture for sensor data traffic in a smart city.

We show, in Figure 1, a general system-architecture for implementing the communication in a smart city using a single ubiquitous, portable and easily deployable in different contexts mesh-network. We consider accesses from sensing sources linked to any MAP to Internet or any output device.

Our ubiquitous and portable network is composed of a set of MAPs with at least WiFi connectivity to the other MAPs. Although any routing algorithm could be used [39,40,41], OLSR is our candidate (as explained in Section 2) because it has been tested for heterogeneous traffic in MANETs and WMNs. One of the MAPs could have external connectivity, e.g., a wired connection or through a conventional radio access network like General Packet Radio Service (GPRS) or Long Term Evolution (LTE). These MAPs will be considered communication sinks.

**Figure 2:**
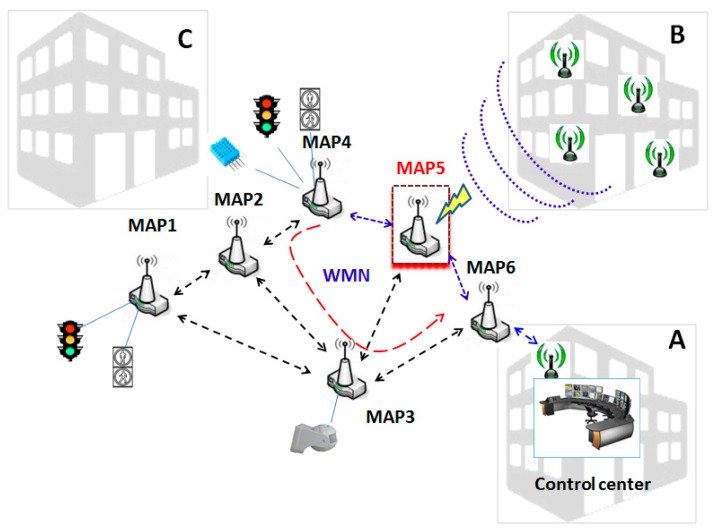
MAP5 is being interfered generating service disruptions due to traffic from building B.

It is a challenging task to develop a proposal for improving the behaviour of any wireless network towards external interferences. We focus our approach on wireless networks which only support reduced or relatively low-rate sensor data traffic. This simplifies MAPs operation, especially in connectivity, packet routing, and managing their wireless interface activation and deactivation. Figure 2 illustrates the specific approach which we will follow. It shows various data sources from different sensors. Likewise, it can be observed that several external interferences are influencing MAP5 and this could cause adverse effects on the traffic supported by the routes which use this node that we want to redress. Several measures made in real networks [42] and extended in Section 6 verify the interfering effects among different networks sharing the same technology.

Figure 2 illustrates how these interferences can affect any MAP in a PWMNS. MAP5 is being interfered by the WiFi devices from building B while it is being used to transport packets between MAP6 and MAP4. Our mechanism will intend to redress these interferences by setting MAP5 wireless interface down. This mechanism will also be applied to any other MAP which are not being used in any route, in order to increase the PWMNS power saving. Although the interference problem can also be solved using other different techniques such as load balancing [43], or multipath routing [44]; we focus on it from a different point of view. Furthermore, unlike [43,44], we also adopt the objective of achieving a certain level of power saving.

## 4. The Up/Down Mechanism of WiFi MAPs

In this Section we describe a proposal for redressing the effects caused by external interferences on MAPs as well as simultaneously reducing power consumption. We consider that, under certain interfering conditions, a MAP could exclude itself from its PWMNS. Specifically, the affected MAP would request the PWMNS to take its wireless interface down. It is important to remark that we do not propose turning the MAP completely off, but only deactivating its wireless interface to the PWMNS. This action also involves power savings as well as a possible traffic rerouting.

### 4.1. State Diagram

In Table 1, we define the different states we will consider on any MAP from the power consumption point of view. Under the *MAP_OFF_s* state, the MAP does not have any consumption; but will also not be able to autonomously reactivate itself to participate in any PWMNS route again. In an *IFACE_DOWN_s* state, the MAP is saving the power required to communicate through its wireless interface; but will be able to set its wireless interface up at any time. Finally, under *MAP_ON_s* state, the MAP is consuming both the power required for its autonomous operation and its wireless interface to transmit and receive.

In compliance with both our power saving and interference redressing targets, MAPs in *MAP_ON_s* state (under certain conditions) will change their state to *IFACE_DOWN_s*. However, MAPs cannot be in this state for a long time, because they can be necessary as alternative routes in the PWMNS and, furthermore, the interferences’ effects could have disappeared.

We propose that there should be a compromise between node availability and consumption. Regarding the power savings involved in this process, they depend on that amount of time spent in *IFACE_DOWN_s* state. Hence, in addition to only considering the wireless interface deactivation when interferences appear, we also suggest applying our proposal in [25]; in order for the intermediate nodes (neither source nor sink) to repeatedly change from *MAP_ON_s* to *IFACE_DOWN_s* states. Thus the power savings will be greater than if only applied when those nodes are interfered.

**Figure 3:**
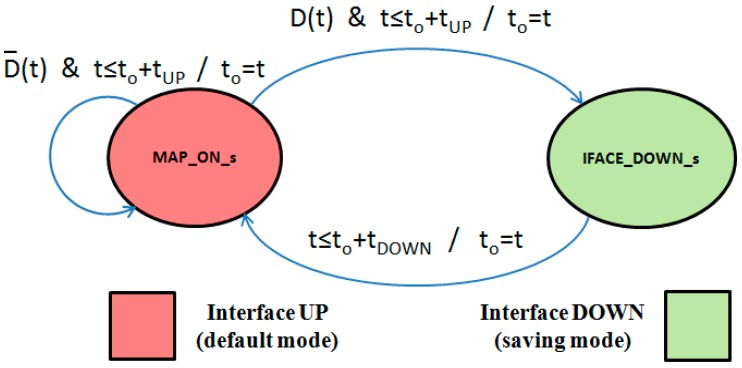
State diagram for our proposal.

After a node enters an *IFACE_DOWN_s* state it must return to the *MAP_ON_s* state, so that the node can check if it is required in any route. Hereinafter, we will refer this node as an inactive node; in contrast to any other node maintaining *MAP_ON_s* state, which we will refer as active node. Those modes of operation are illustrated in the state diagram of Figure 3. It shows two states corresponding to the 2nd and 3rd entries in Table 1. It is also indicated the conditions and exit values for every transition in the form condition/variable = exit_value. We must highlight the Boolean downability conditioning-variable D(t), which indicates whether the node will become inactive at instant $t_{o}\;+\;t_{UP}$ or not. Hence, it is possible to transition from *IFACE_DOWN_s* state to *MAP_ON _s* state when $t_{DOWN}$ seconds have elapsed since reference instant $\;t_{o}$; and then $t_{o}$ will be updated to the current instant. From the *MAP_ON _s* state both possible transitions require $t_{UP}$ seconds to have elapsed since the $t_{o}$ reference instant (and then $t_{o}$ will be updated to the current instant), but if the node is downable, i.e., D(t) = true, it must transition to the *IFACE_DOWN_s* state; and otherwise, i.e., D(t) = *false*, the node is to be used in any route and so it will remain in its *MAP_ON_s* state.

**Figure 4:**
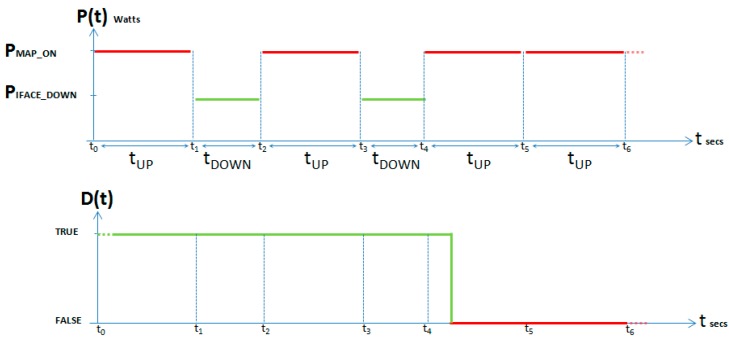
Power consumption variation related to D(t).

In Figure 4 we illustrate an example of the time evolution of the average power P(t) consumed by some node, related to the time evolution of the downability variable D(t) corresponding to the same node, according to the state diagram of Figure 3. Thus while D(t) remains true, its P(t) changes repeatedly between P_MAP_ON_ Watts for $t_{UP}$ seconds and P_IFACE_DOWN_ Watts for $t_{DOWN}$ seconds. When D(t) changes to false (after $t_{4}$ instant) P(t) will remain at P_MAP_ON_ Watts level, and this value will be repeated while D(t) remains false.

Let $P_{\Delta t}$ be the average power consumed by each MAP for an Δt interval in the PWMNS, and $C_{\Delta t}$ be the power consumption per unit of time. The average power $P_{\Delta t}$ is calculated by Equation (1) assuming that, for a time interval $\Delta t\;=\;m\;\times \;t_{DOWN}\;+\;n\;\times \;t_{UP}$, *IFACE_DOWN_s* state is reached *m* times and *MAP_ON_s* state *n* times:

(1) $$P_{\Delta t}\;=\;\;\frac{P_{IFACE\_DOWN}\;\times \;m\;\times \;t_{DOWN}\;+\;P_{MAP\_ON}\times \;n\;\times \;t_{UP}}{\Delta t}$$

while power consumption for the same time interval Δt is calculated by Equation (2):

(2) $$C_{\Delta t}=\;P_{IFACE\_DOWN}\;\times \;m\;\times \;t_{DOWN}+P_{MAP\_ON}\times n\;\times \;t_{UP}$$

We have measured the power consumption in one Linksys [45] MAP working with DD-WRT [46] firmware. Below we show the result, for a day (Δt = 24 h) assuming m = 0 in case 1 and n = 0 in case 2:

1. 9.4 Watts in *MAP_ON_s* (i.e., $C_{\Delta t}\;=9.4\;\mathrm{Watts}\;\times \;24\;h\;=\;225.6\;\mathrm{Watt}\;-\;\mathrm{hour}$).
2. 7.6 Watts in *IFACE_DOWN_s* (i.e., $C_{\Delta t}\;=7.6\;\mathrm{Watts}\;\times \;24\;h\;=\;182.4\;\mathrm{Watt}\;-\;\mathrm{hour}$).

Usual mode for this MAP is case 1, where power saving does not exist. On the other hand, as can be noted in case 2, a maximum power saving of 44 Watts can be achieved for a day of operation. Thus, our mechanism can generate a power saving in the mid-point of both cases, whenever *m* > 0 and *n* > 0.

Power consumption is a kind of human interference in the Environment. According to the Spanish Consumers and Users Organization (OCU) [47], 1 W of power for one year corresponds to 5.7 kg of carbon dioxide emitted to the atmosphere. Making calculations for carbon dioxide savings in the Linksys system, we obtain the following values, respectively:

1. 53.58 kg of carbon dioxide in *MAP_ON_s*.
2. 43.32 kg of carbon dioxide in *IFACE_DOWN_s*.

We compare these results with other recent MAPs, concretely the WRT160NL Router/AP Linksys/Cisco [48] equipped with the standard IEEE 802.11n (two wireless interfaces). Table 2 shows the average power consumed in the considered states.

As can be noted, depending on the higher used-bandwidth, the average power consumed by the MAP is increased by 0.7 or 1.6 Watts, respectively, from the *IFACE_DOWN_s* state. Attending to previous results, they make clear that, when the time interval for the wireless interface to be deactivated is maximized, power savings are also maximized. This deactivation time interval will be increased whenever either *m* or $t_{DOWN}$ duration are increased. Factor *m* is determined by the number of times that the node returns to *IFACE_DOWN_s* state (the node is unused). On the other hand, $t_{DOWN}$ duration impacts the availability of the node in the PWMNS when it is required for any route. We consider $t_{DOWN}$ duration a variable parameter which depends on power saving and traffic requirements. Similarly, $t_{UP}$ duration must also be configurable, because it impacts the speed for the node to be inactive in case it is interfered or just unused.

### 4.2. Basic Ideas on Protocol

As a complement to our idea in [25], in our current proposal we suggest extending it to situations where the node is being used in a route, but interfered. Obviously, measuring external interferences is a quite complex task, and more so to determine their tolerability threshold. Some measurable parameters can be detection of sequence order loss in sensing messages, or excessive delay in their delivering, or non-compliance in expected throughput in the route, among others. In this approach we will assume a generic function κ(t) which models the cited interference parameters.

**Figure 5:**
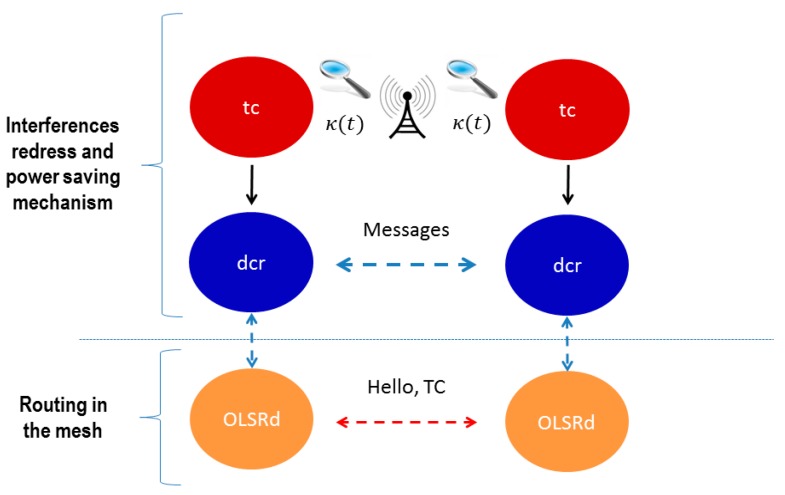
Block diagram for our proposal.

We have to define carefully how our proposal operates in order to change the node state when it is unused or interfered. In this regard, for the first event, we defined in [42,49] a special mechanism of traffic control (we name it tc) implemented in infrastructure WiFi networks APs. In the present proposal we have adapted it for PWMNS, including the ability of redressing interferences. We assume that every MAP can measure its experienced interference level through the previously cited κ(t) function. Based on this function, the MAP has to send a request to change to *IFACE_DOWN_s* to the corresponding neighbours. False alarms do not negatively interfere the routing because the MAP neighbours will or will not confirm and authorize the rerouting of the traffic. In Figure 5 we show how dcr and tc can work together in a MAP taking into account κ(t). Thus dcr is adapted to implement this new feature of redressing interferences and power saving. It must be clear that we do not propose any change on OLSR, but influencing its decisions with our mechanism. Figure 5 also includes a set of messages in order to communicate dcr from different neighbours as well as standard OLSRd messages. These messages are described in Table 3.

We consider that any MAP which fulfils the requirements for entering *IFACE_DOWN_s* state is a downable MAP (hereinafter dMAP). Let us explain how the communication with the dMAP neighbours is managed in a general case. The direct neighbours of the dMAP, using dcr, need to know the *t_DOWN_* set up by this latter. Thus, every dMAP must inform its neighbours about its *t_DOWN_*, just before it changes to *IFACE_DOWN_s*. This can be done by sending a control message (we name it *GO_IFACE_DOWN*) containing also its identification (*id_*MAP). Thus, all the neighbours are informed when the dMAP changes again to *MAP_ON_s*. Before the dMAP changes to *IFACE_DOWN_s*, it needs to be confirmed by all of its neighbours, in order for the former not to lose data coming from the latter ones. This is accomplished by forcing the dMAP to wait for acknowledgements (*ACKs*) from all its neighbours. The *t_DOWN_* negotiation information is included as part of the ACK message. Though *t_DOWN_* duration is initialized to a default value in the dMAP, it can be modified after ACK interchange, selecting the minimum returned value. It is also necessary for the dMAP to receive a negative acknowledgement (NACK), in case any neighbour needs the participation of the dMAP in any route. In both cases, the dMAP will wait until a programmable timeout will expire. In Table 3 we define the messages including the name of the messages, its arguments, the direction of communication and a brief description. In this table the initiator is any dMAP that requests to its neighbours confirmation and authorization for setting down its wireless interface.

### 4.3. Algorithm Specification

In Algorithms 1–4, we show the pseudocode of our proposal for tc and dcr, respectively, which concurrently run in every MAP. As regards tc, once an interference level threshold θ is defined (line #1), this algorithm will continuously sense the last interference level *κ* (line #3) and query for two conditions (line #4): if κ exceeds the defined threshold θ or the node is unused. According to these conditions, tc sends asynchronously the last MAP previous-downability value Dp to dcr (line #9). It is possible for Dp to change while dcr processes it, but we mainly expect our algorithms to handle properly dMAPs interfered or unused for a long time.

**Algorithm 1:** Basic Pseudocode of tc1: Define θ 2: **Repeat** 3: **Sense** (κ) 4: **if** (κ > θ or MAP is unused) **then** 5: Dp = true 6: **else** 7: Dp = false 8: **endif** 9: **Send** (Dp) **to** dcr 10: **Forever**

Simultaneously, the dcr main process (Algorithm 2) will continuously update the reference starting time *t_o_* (lines #3–#4) and, in the case that MAP was in *MAP_ON_s* state (lines #5–#6), two timed threads will be launched for *t_o_* + *t_UP_* seconds (line #7). Both threads will be stopped after the selected time, and a definitive downability value D will be returned, according to the previous downability Dp (tc line #9). If MAP is definitively downable, its state will be changed to *IFACE_DOWN_s* and its wireless interface will be deactivated (lines #8–#12). Finally, in the case that MAP was in *IFACE_DOWN_s* state (line #12), it will wait for *t_o_* + *t_DOWN_* seconds (line #13), will activate its wireless interface (line #14), and will change its state to *MAP_ON_s* state (line #15).

Algorithm 3 shows our proposed pseudocode for dcr dMAP_proc process launched from dcr main process line #7. It always initializes MAP downability D to false which will be the default value returned to dcr main process when either the rest of dMAP_proc does not modify this value, or the thread is stopped by the programmed timer in dcr main process line #7. The former condition is tested after receiving the last Dp from tc (lines #2–#3). If this previous-downability is met, the MAP will send a *GO_IFACE_DOWN* message to its neighbours (line #4) and will wait for receiving their confirmation or a timeout (line #5). Then, if an ACK is received from all neighbours (line #6), will send a DOWN message to its neighbours (line #7), will update its *t_DOWN_* timer as the minimum it received from its neighbours (line #8), and will return a true value for D (line #9).

**Algorithm 2:** Basic Pseudocode of the dcr Main Process1: Define MAP_state = *MAP_ON_s*, *t_UP_*, *t_DOWN_* 2: **Repeat** 3: **Read** (*t*) 4: *t*_o_ = *t* 5: **Switch** (MAP_state) 6:  **case**
*MAP_ON_s*: 7: **timedThread** (*t_o_* + *t_UP_*, dMAP_proc, neighbour_proc) 8: **if** (D) **then** 9: **set** Wireless Interface **DOWN** 10: MAP_state = *IFACE_DOWN_s* 11: **endif** 12:  **case**
*IFACE_DOWN_s*: 13: **Wait** (*t_o_* + *t_DOWN_*) 14: **set** Wireless Interface **UP** 15: MAP_state = *MAP_ON_s* 16: **endSwitch** 17: **Forever**

**Algorithm 3:** Basic Pseudocode of the dcr dMAP_Proc Process**timedThread** dMAP_proc 1: D = false 2: **Receive** (Dp) **from**
*tc* 3: **if** (Dp) **then** 4: **Send** (*GO_IFACE_DOWN*) **to** neighbours’ dcr 5: **Receive** (confirmation, *t_DOWN_*, timeout) **from** neighbours’ dcr 6: **if** (confirmation = ACK and not timeout) **then** 7: **Send**(DOWN) **to** neighbours’ dcr 8: *t_DOWN_* = min(*t_DOWN_* received from neighbours’ dcr) 9: D = true 10: **endif** 11: **endif** **endtimedThread**

**Algorithm 4:** Basic Pseudocode of the dcr Neighbour_Proc Process**timedThread** neighbour_proc 1: **Receive** (*GO_IFACE_DOWN*) **from** id_MAP 2: **if** (id_MAP is used in a route for any sink and not alternative route for that sink) **then** 3: **Send** (NACK) **to** id_MAP 4: **else** 5: **Evaluate** (*t_DOWN_*) **for** id_MAP 6: **Send** (ACK, *t_DOWN_*) **to** id_MAP 7: **endif** 8: **Receive** (confirmation, timeout) **from** id_MAP 9: **if** (confirmation = DOWN and not timeout) **then** 10: **UpdateDB** (delete any route which includes id_MAP) 11: **end** **endtimedThread**

Algorithm 4 shows our proposed pseudocode for a dcr neighbour_proc process launched from dcr main process line #7. We must remember it will stop once thread timer is elapsed (dcr main process line #7). Firstly, it will wait for receiving any *GO_IFACE_DOWN* message for any dMAP (line #1). Then, if the considered dMAP is irreplaceable (line #2), this neighbour_proc will send a NACK to it (line #3). Otherwise, a maximum *t_DOWN_* timer will be evaluated (line #5) in order to be sent, along with an ACK, to the considered dMAP (line #6). In either case (ACK or NACK) this neighbour_proc process will wait for receiving a DOWN confirmation (or a timeout) from the dMAP (line #8). If a DOWN message is received (line #9) this neighbour_proc will then update its routing table by deleting dMAP from it (line #10).

**Figure 6:**
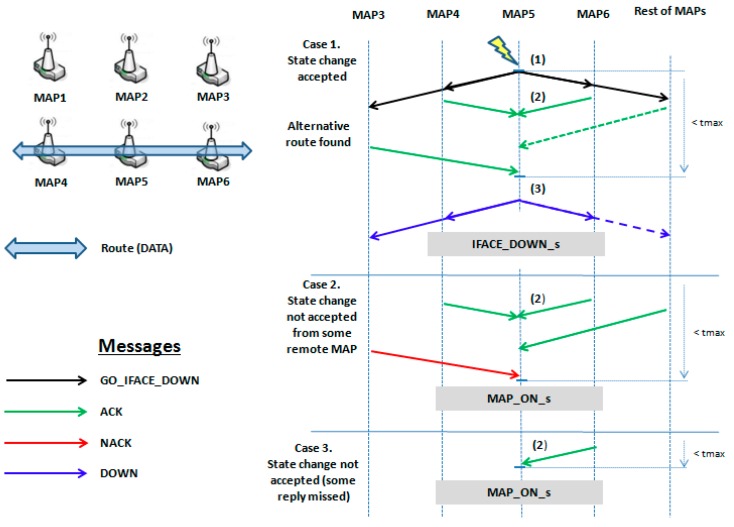
Sequence of messages and state changes for MAP5.

Continuing the example in Figure 2, we show in Figure 6 the sequence of actions to follow under our mechanism. Initially, there is a communication route among MAP4, MAP5 and MAP6 (marked as a blue wide arrow). The instant when the tc of MAP5 detects interferences is marked with (1). Then, at instant marked with (2), MAP5 requests to change to *IFACE_DOWN_s*, by sending (black arrows) the message named *GO_IFACE_DOWN* (MAP5 dcr will send it to its neighbours’ dcr) and it will wait for their answers.

All neighbours, before returning an answer, will analyze their routing tables or query the routing protocol to find another route to the destination (actions performed by the OLSRd). All neighbours which are granted an alternative route will reply with an ACK message (green arrow). If any neighbour did not obtain its replacement route, it should reply with a NACK message (red arrow). Then, once MAP5 receives all ACKs, it broadcasts a last message, named DOWN (blue arrows), as notification for closing the process. At that instant, marked with (3), MAP5 changes to *IFACE_DOWN_s* and MAP4 will redirect its traffic through another MAP.

We show, in case 2, the situation in which one MAP replies NACK (red arrow). In this case MAP5 stays in *MAP_ON_s* state. Finally, in case 3 we show that if some answer is lost, MAP5 will also stay in *MAP_ON_s* state after a programmable timeout tmax.

## 5. Interference Redress and Power Saving Factors

It is important to have a measure for the interference redress and power savings achieved by our mechanism. This requires knowing how much the extra average power consumed by an active MAP is, related to when it is inactive.

### 5.1. Mathematical Model

Let $P_{m,n}^{MAP\_ON}$ be the average power consumed by a MAP with its wireless interface is set up, and $P_{m,n}^{IFACE\_DOWN}$ when it is set down. Similarly, given an inactive MAP, let $t_{DOWN}$ be the time interval during which it sets its wireless interface down, and $t_{UP}$ the time interval during which it sets it up, in order to check for its requirement in any route. Equation (3) defines the extra average power $E_{m,n}$ consumed by the MAP when it is active respect to $R_{m,n}$, which is what it consumes when is inactive for a cycle $\Delta t\;=\;t_{DOWN}\;+\;t_{UP}$:

(3) $$E_{m,n}\;=\;P_{m,n}^{MAP\_ON}\;-\;R_{m,n}\;=\;\;P_{m,n}^{MAP_{ON}}\;-\;\frac{P_{m,n}^{IFACE_{DOWN}}\cdot \;t_{DOWN}\;+\;P_{m,n}^{MAP_{ON}}\cdot \;t_{UP}}{t_{DOWN}\;+\;t_{UP}}\;=\frac{\;\left(P_{m,n}^{MAP\_ON}\;-\;P_{m,n}^{IFACE\_DOWN}\right)\cdot \;t_{DOWN}}{t_{DOWN}\;+\;t_{UP}}$$

At this point we are able to measure the average power consumed by any MAP. Thus, let $A_{m,n}\left(t\right)$ be a matrix representing the active MAPs of our PWMNS at instant *t*. Let Z be any of the possible source-sink pairs to be communicated through our PWMNS. If we define $\gamma_{m,n}^{Z}$ as node matrix which must be active in order to establish the route able to communicate the given Z consuming the minimum average power possible, and $\Gamma_{m,n}^{Z}$ that of maximum average power, Equation (4) determines which power saving $S^{Z}\left(t\right)$ will be the reached if the current set of active nodes at instant *t* is $A_{m,n}\left(t\right)$:

(4) $$S^{z}(t)=\frac{\sum_{m=1}^{M}\sum_{n=1}^{N}\left\{R_{m,n}\;+\;E_{m,n}\;\times \Gamma_{m,n}^{Z}\right\}\;-\;\sum_{m=1}^{M}\sum_{n=1}^{N}\left\{R_{m,n}\;+\;E_{m,n}\;\times \;A_{m,n}\left(t\right)\right\}}{\sum_{m=1}^{M}\sum_{n=1}^{N}\left\{R_{m,n}\;+\;E_{m,n}\;\times \Gamma_{m,n}^{Z}\right\}\;-\;\sum_{m=1}^{M}\sum_{n=1}^{N}\left\{R_{m,n}\;+\;E_{m,n}\;\times \;\gamma_{m,n}^{Z}\right\}}\;=\frac{\sum_{m=1}^{M}\sum_{n=1}^{N}\left\{E_{m,n}\;\times \Gamma_{m,n}^{Z}\right\}\;-\;\sum_{m=1}^{M}\sum_{n=1}^{N}\left\{E_{m,n}\;\times \;A_{m,n}\left(t\right)\right\}}{\sum_{m=1}^{M}\sum_{n=1}^{N}\left\{E_{m,n}\;\times \Gamma_{m,n}^{Z}\right\}\;-\;\sum_{m=1}^{M}\sum_{n=1}^{N}\left\{E_{m,n}\;\times \;\gamma_{m,n}^{Z}\right\}}$$

Now we will define a measure for evaluating the degree of interference redress of our mechanism. Let $\kappa_{m,n}\left(t\right)$ be the interference experienced by every MAP at instant *t*. If we define $\rho_{m,n}^{Z}$ as the matrix of active nodes configuring the route with maximum interference level able to communicate the given Z, then Equation (5) determines its corresponding interference redress level $R^{Z}\left(t\right)$ reached when the set of current active nodes is $A_{m,n}\left(t\right)$. It should be noted that, unlike Equation (4), in this case the minimum level is always set to 0. Otherwise, if every node was equally interfered, the factor $R^{Z}\left(t\right)$ would return the maximum value ($R^{Z}\left(t\right)\;=\;1$) which would indicate an optimum interference redress, while we would actually like to alert about any interference, even if it is the minimum. This is a different criterion respect to power saving factor $S^{Z}\left(t\right)$ because, in this latter, there will always exist an extra average power consumed $E_{m,n}\;>\;0$; and our target, in this case, is that $S^{Z}\left(t\right)\;=\;0$ indicates the route for Z which minimizes the overall extra average power consumed:

(5) $$R^{Z}\left(t\right)\;=\;\frac{\sum_{m=1}^{M}\sum_{n=1}^{N}\left\{\kappa_{m,n}\left(t\right)\;\times {\;\rho }_{m,n}^{Z}\right\}\;-\;\sum_{m=1}^{M}\sum_{n=1}^{N}\left\{\kappa_{m,n}\left(t\right)\;\times \;A_{m,n}\left(t\right)\right\}}{\sum_{m=1}^{M}\sum_{n=1}^{N}\left\{\kappa_{m,n}\left(t\right)\;\times {\;\rho }_{m,n}^{Z}\right\}}$$

Therefore, we consider that the desirable condition for our PWMNS is characterized by $R^{Z}\left(t\right)\;\approx \;1$, but keeping $S^{Z}\left(t\right)\;\approx \;1$; which implies a high degree of interference redress, but preserving a power saving close to the optimum.

### 5.2. Simulation Results for Power Saving and Interference Redress Factors

**Figure 7:**
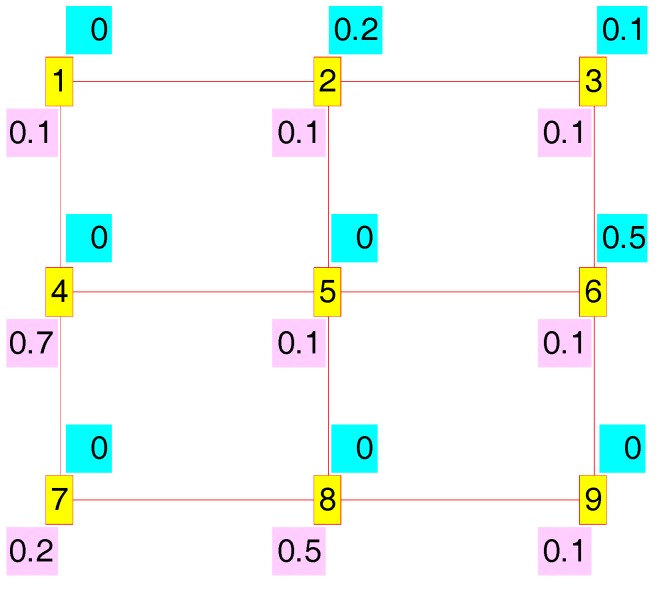
Regular 3 × 3 mesh with city block routes.

In order to clarify the meaning of these measures we will use a straightforward example consisting of a regular 3 × 3 mesh which is illustrated in Figure 7. In this example we will assume as condition that the nodes are separated the maximum coverage distance. As we showed in our work in [26], this condition implies that the routes can only follow the city block geometry, i.e., every node can only establish direct routes with its left, right, up or down neighbours. In Figure 7, every node is labelled with its identifier (a number from 1 to 9 inserted in a red box with yellow background), its $E_{m,n}$ (a value on cyan background over the identifier) and its $\kappa_{m,n}$ (a value on purple background down the identifier). The routes established among the nodes are indicated by a red line, so that in Figure 7 all the possible direct routes are established.

**Figure 8:**
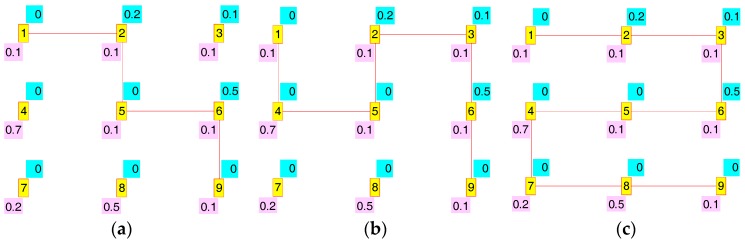
(**a**) S(t) = 1, R(t) = 0.1; (**b**) S(t) = 0.5, R(t) = 0; (**c**) S(t) = 0, R(t) = 0.

Following we will study the power saving and interference redress possibilities which result when we establish node 1 as source and node 9 as sink. Figure 8 shows some of the routes which give: (a) one of the maximum power saving cases, concretely S(t) = 1, $\;\sum_{m=1}^{M}\sum_{n=1}^{N}\left\{E_{m,n}\;\times \;A_{m,n}\left(t\right)\right\}\;=\;0.5$, R(t) = 0.1, and $\;\sum_{m=1}^{M}\sum_{n=1}^{N}\left\{\kappa_{m,n}\;\times \;A_{m,n}\left(t\right)\right\}=0.7$; (b) one of the intermediate cases, corresponding to S(t) = 0.5, $\;\sum_{m=1}^{M}\sum_{n=1}^{N}\left\{E_{m,n}\times A_{m,n}\left(t\right)\right\}\;=\;1.3$, R(t) = 0, and $\;\sum_{m=1}^{M}\sum_{n=1}^{N}\left\{\kappa_{m,n}\;\times \;A_{m,n}\left(t\right)\right\}=0.8$; and (c) one of the routes with the minimum power saving, specifically S(t) = 0, $\;\sum_{m=1}^{M}\sum_{n=1}^{N}\left\{E_{m,n}\;\times \;A_{m,n}\left(t\right)\right\}\;=\;2$, R(t) = 0, and $\;\sum_{m=1}^{M}\sum_{n=1}^{N}\left\{\kappa_{m,n}\;\times \;A_{m,n}\left(t\right)\right\}\;=\;0.8$. In all cases more than one route gave the same level of S(t).

**Figure 9:**
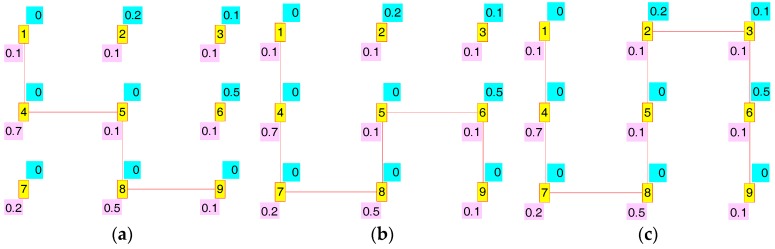
(**a**) R(t) = 1, S(t) = 0.3; (**b**) R(t) = 0.4, S(t) = 0.1; (**c**) R(t) = 0, S(t) = 0.

Figure 9 illustrates some of the results for the interference redress R(t) study: (a) one of the routes with the maximum redress, concretely R(t) = 1, $\;\sum_{m=1}^{M}\sum_{n=1}^{N}\left\{\kappa_{m,n}\;\times \;A_{m,n}\left(t\right)\right\}\;=\;0$, S(t) = 0.3, and $\;\sum_{m=1}^{M}\sum_{n=1}^{N}\left\{E_{m,n}\;\times \;A_{m,n}\left(t\right)\right\}\;=\;1.5$; (b) one with an intermediate value, corresponding to R(t) = 0.4, $\;\sum_{m=1}^{M}\sum_{n=1}^{N}\left\{\kappa_{m,n}\;\times \;A_{m,n}\left(t\right)\right\}\;=\;0.5$, S(t) = 0.1, and $\;\sum_{m=1}^{M}\sum_{n=1}^{N}\left\{E_{m,n\;}\times \;A_{m,n}\left(t\right)\right\}\;=\;1.8$; and (c) one of the routes with the minimum redress factor, specifically R(t) = 0, $\;\sum_{m=1}^{M}\sum_{n=1}^{N}\left\{\kappa_{m,n}\;\times \;A_{m,n}\left(t\right)\right\}\;=\;0.8$, S(t) = 0, and $\;\sum_{m=1}^{M}\sum_{n=1}^{N}\left\{E_{m,n}\;\times \;A_{m,n}\left(t\right)\right\}\;=\;2$.

Though routes shown in Figure 8 and Figure 9 are different, there are other routes which share their same S(t) and R(t) level. Thus Figure 8c and Figure 9c are both valid for S(t) = 0 (minimum power saving) and R(t) = 0 (minimum interference redress factor). On the other hand, we can also appreciate the usefulness of our factors in adjusting to a unitary range the highly-uneven original range of the corresponding measures $\sum_{m=1}^{M}\sum_{n=1}^{N}\left\{\kappa_{m,n}\;\times \;A_{m,n}\left(t\right)\right\}$ and $\sum_{m=1}^{M}\sum_{n=1}^{N}\left\{E_{m,n}\;\times \;A_{m,n}\left(t\right)\right\}$. This is better illustrated in Table 4, where we show the data generated by all possible routes.

One of the issues facing our proposal is the simultaneous reachability of our both targets: power saving and interference redress. In Table 4 we can observe that those targets are not always incompatible, so that in the 3rd entry (route 1-2-5-8-9) a high value has been reached for both factors: S(t) = 0.7 and R(t) = 0.8.

In order to verify how frequently we can reach both targets, we made a new simulation in a 3 × 3 mesh where we set $E_{m,n}\;=\;0.1\;W$ for all nodes, and generated all the possible combinations of binary interferences $\kappa_{m,n}$. It must be clear that we have only considered binary levels of interference, i.e., $\kappa_{m,n}\;=\;\left\{0|1\right\}$, where 0 means not interfered and 1 interfered.

**Figure 10:**
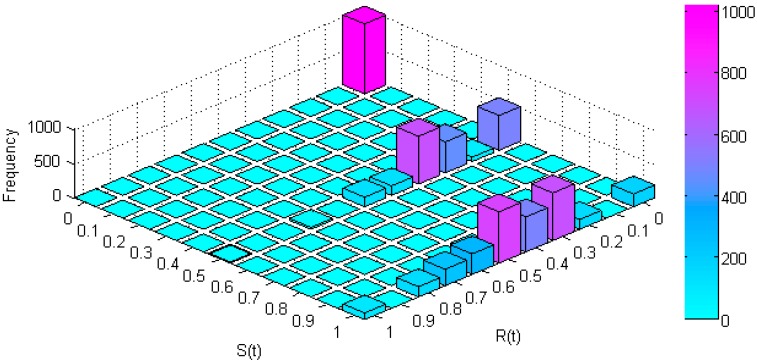
Relationship between R(t) and S(t) in a 3 × 3 PWMNS.

On the other hand, though $E_{m,n}$ is fixed to a constant value for every node, extra average power varies according to the length of the considered route. Figure 10 shows the resulting histogram for R(t) vs. S(t) considering all the possible routes in the described conditions. As we can observe the highest peak is placed at {R(t),S(t)} = {0,0}, which corresponds to the longest routes which in addition crosses the most interfered nodes.

We can also appreciate that the combination {R(t),S(t)} = {1,1} seldom occurs (but it does). However we can highlight a significant combination for 0.5 < R(t) < 1 and 0.5 < S(t) < 1, which represents favourable cases for both power saving and interference redress, simultaneously. We should not forget that $E_{m,n}$ is fixed to a constant value for every node, so the S(t) values are inversely proportional to routes length. In fact, Figure 10 shows three clearly visible groups (0, 0.5 and 1) for S(t), which correspond to the three groups of route length showed in Table 4 (5, 7 and 9 nodes).

**Figure 11:**
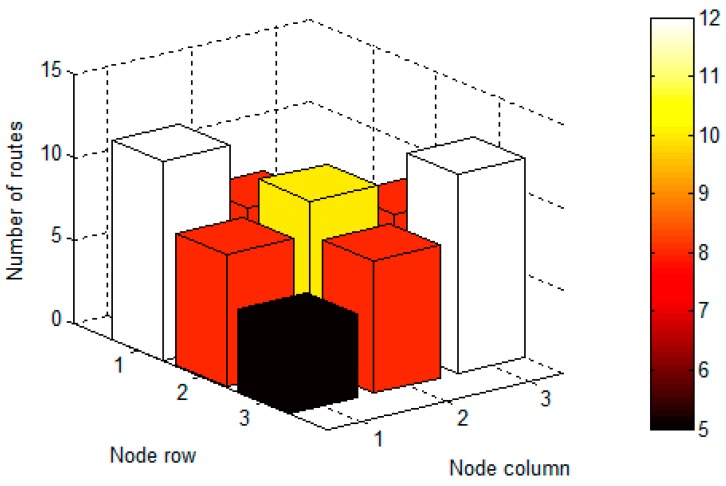
Number of routes passing through each node in a 3 × 3 PWMNS.

It seems obvious that interference impact is higher in central nodes of PWMNS. Anyway it is clearly illustrated in Figure 11 which shows (in the *Z*-axis) the number of routes crossing every node of the 3 × 3 mesh. Excepting the source (1,1) and sink (3,3), the next more visited node is the centre (2,2), being the less visited those of the remaining corners (1,3) and (3,1).

**Figure 12:**
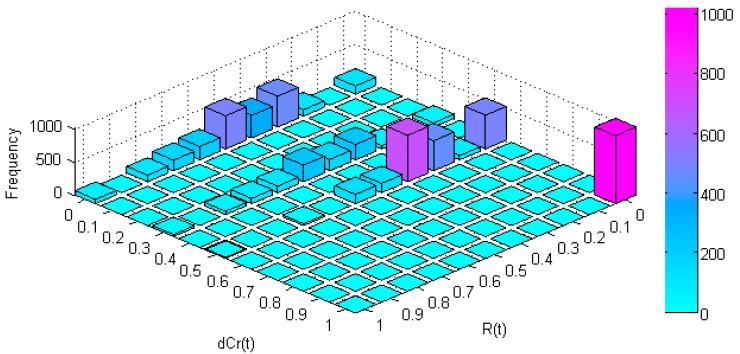
Relationship between R(t) and node proximity to the centre in a 3 × 3 PWMNS.

We will analyze in more depth this relationship between interference influence and interfered node position into the mesh. For this purpose we defined the function *dCr*(*t*) which measures the proximity of the nodes belonging to the considered route *r* at instant *t* relative to the centre of the mesh. This function sums the mesh centre proximity of every route node and scales it between 0 (the route with less centred nodes) and 1 (the route with more centred nodes). The result is illustrated in Figure 12.

Figure 12 shows that, for a centre proximity *dCr*(*t*) < 0.5, the interference redress returns values along all its definition range. However, when the interference appears in nodes very close to the mesh centre (*dCr*(*t*) > 0.5) the interference redress falls to R(t) = 0. The variability of centre proximity *dCr*(*t*) is very limited for the routes we are considering (Table 4). For this reason its values are distributed in only four groups.

## 6. Experimental Results for a Test Platform Based on Raspberry Pi

In this Section we will analyze the results of several experiments implemented on a PWMNS based on RPi.

### 6.1. Interface Deactivation and External Traffic Effects

**Figure 13:**
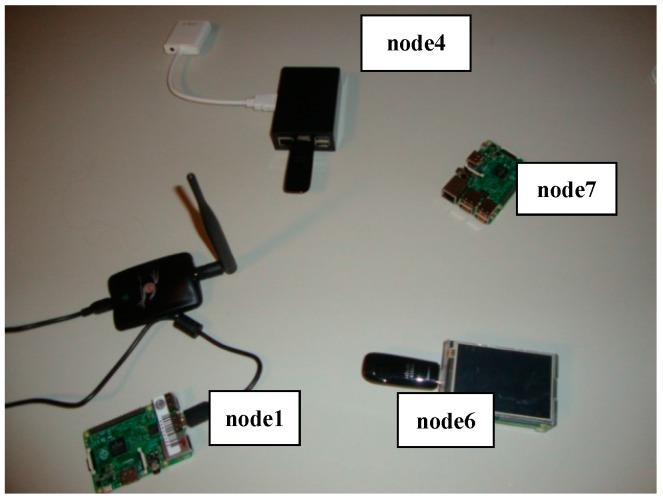
Image of our test platform.

In order to analyze the behaviour of a real PWMNS, we have assembled a test platform equipped with four RPi MAPs (named node 1, 4, 6 and 7 in Table 5 and Figure 13). We have chosen these devices due to their powerful processing capability, multiple USB I/O and GPIO, and specially their versatile and complete Operative System (based on UNIX/Linux).

**Figure 14:**
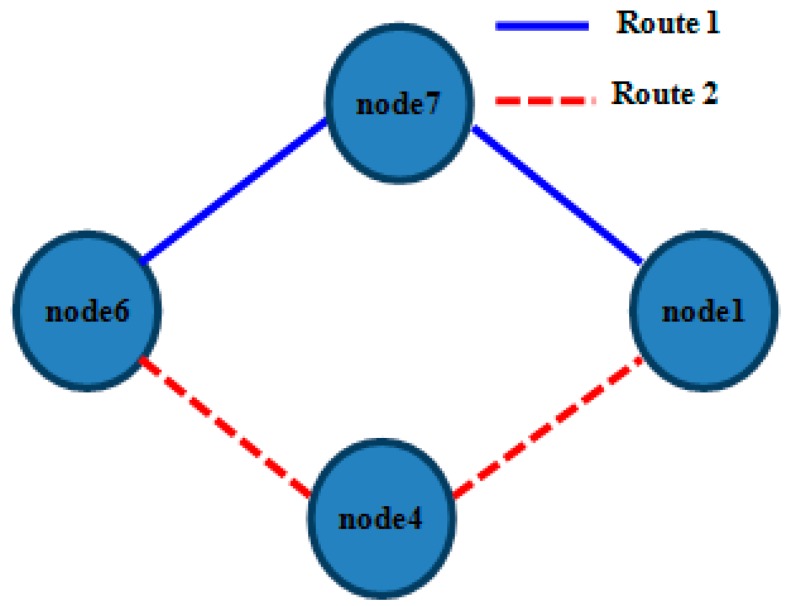
Deployed static topology.

All nodes established their WiFi ad-hoc communication on channel 7 and OLSR to achieve a mesh configuration. We have deployed them in a static indoor placement, in order for them to establish a link structure similar to what is showed in Figure 14. This topology allows node6 (which was used as data source) to be able to communicate using any intermediate nodes (node7 or node4) to node1 (which was used as data sink). We used four RPis because we wanted to show that, with a reduced amount of redundant paths, our strategy worked properly. With greater number of redundant paths (more RPis) our strategy will continue to work; because a MAP will only set its WiFi interface down when its neighbours acknowledge it. Direct communication from node6 to node1 was seldom established, due to deployment conditions. The chosen route was decided by OLSR based on its operation mode, and interchanged configuration messages and metrics.

The IP addresses assigned to the nodes were 192.168.200.6 (for node6), 192.168.200.4 (for node4), 192.168.200.7 (for node7) and 192.168.200.1 (for node1). All these addresses were statically fixed in order to make the addressing task easier and avoid their changes during the test. Focusing on what happened in node6, we show in Table 6 the most habitual state for its routing table.

This routing table provides direct connectivity for node7 and node4. But, in order to reach node1, this table provides two possible routes: route1 (node6-node7-node1) and route2 (node6-node4-node1). As may be seen, the static topology guarantees that node6 will use one of the cited routes in order to communicate with node1 as well as there will always exist a direct connection between node6 and both node4 and node7, so that any of the latter can be used as intermediate node for the former to reach node1. Obviously we also guarantee that node1 has a direct link to both node4 and node7.

**Figure 15:**
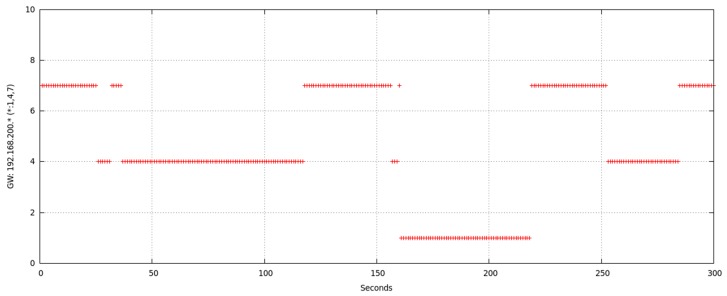
Chosen intermediate node for route from node6 to node1.

Figure 15 shows the time evolution of the chosen intermediate node for reaching node1 based on successive readings of node6 routing table. Those readings were made during several sessions of 300 s leaving a 1 s interval between every pair of readings. Data were obtained without varying any configuration or traffic aspect. Vertical axis represents the following node (after node6) in the route; so that possible values were node7, node4 and directly node1. In view of Figure 15 we must highlight that the direct route from node6 to node1 was seldom chosen.

We can detect that node6 uses as intermediate node mainly both the node7 and node4 to reach node1. Though both are valid, distance and other environmental conditions (radio channel instability and especially typical variation [50] of WiFi signal) cause OLSR to change dynamically the chosen route.

As OLSR chose a single route among all the possibilities in order to establish connectivity between every pair of nodes, we considered that every node not participating in any route was susceptible for deactivating its wireless interface, so that it could contribute to power saving and avoid possible interferences. In order to check our test platform behaviour under interferences and the validity of this proposal, we have used several applications which generate some data traffic on the route node6-node1. We specifically used traffic which emulates the following two patterns:

- Connection-less Low Frequency Traffic (LFT) with Internet Control Message Protocol (ICMP).
- Connection-oriented High volume and Frequency previously stored Traffic (HFT) with Secure Copy (scp).

#### 6.1.1. Effect of WiFi Interface Deactivation

**Figure 16:**
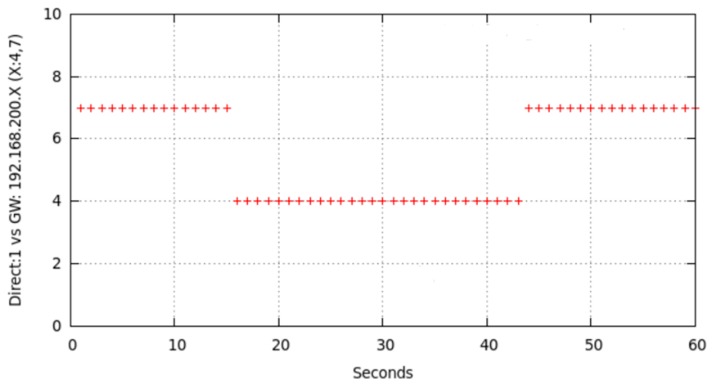
Deactivation effect of intermediate node7 WiFi interface (t_DOWN_ = 2 s) at instant *t* = 10.

Figure 16 illustrates the result of one of the multiple tests performed in order to check what happens in node6 routing table when node7 (one of the intermediate nodes) interface was deactivated for 2 s in a 60 s session. Specifically, initial chosen route was node6-node7-node1. At instant *t* = 10 we deactivated node7 WiFi interface for 2 s and, after this time was elapsed, we reactivated node7 WiFi interface again until *t* = 60. We repeated this experiment changing the interface deactivation interval. Similarly we checked our proposal of implementing a state “duty cycle”, i.e., cyclically repeated changes on the state of node from *MAP_ON_s* to *IFACE_DOWN_s*. The obtained results prompt us to state that, for this cyclical process, small t_DOWN_ values were not optimal, especially for medium or high data-rate traffic. This was due to OLSR definitively deletes that route from the table, because of its high variability. With an adequate selection of t_DOWN_, the right rerouting (through node4) later undone (and recovered through node7) is evidenced in Figure 4. Obviously, our mechanism could be applied on any unused node that, as node4, was not participating in any route (or is being interfered); because OLSR could find a right substitute and even could reuse the inactive node whenever possible.

We can deduct from Figure 16 that node7 was initially in the chosen entry, as intermediate node (“gateway”), in node6 routing table for reaching node1. At *t* = 10, node7 changed to *IFACE_DOWN_s* state. The cited routing table was not immediately modified, but it waited for an updating time related to OLSR configuration. This time length was variable and associated to the time length during which OLSR waited for messages from node7 or other nodes announcing they detected to node7. In this experiment, as these messages were not received, OLSR searched for another route in order to reach node1, it found node4 and updated the corresponding entry in the table. That updating time-length was related to time intervals configured for propagating topology changes. In our experiment, five seconds were elapsed for changing the table with the new route. After that, for approximately 30 s the new entry remains in the table. Though at *t* = 12 node7 was *MAP_ON_s* again and announcing itself, and node6 was receiving its announcements, OLSR did not change the table in order to reuse node7 until approximately *t* = 44. We have checked and analyzed these messages through simultaneously use of *tcpdump* for capturing network traffic.

**Figure 17:**
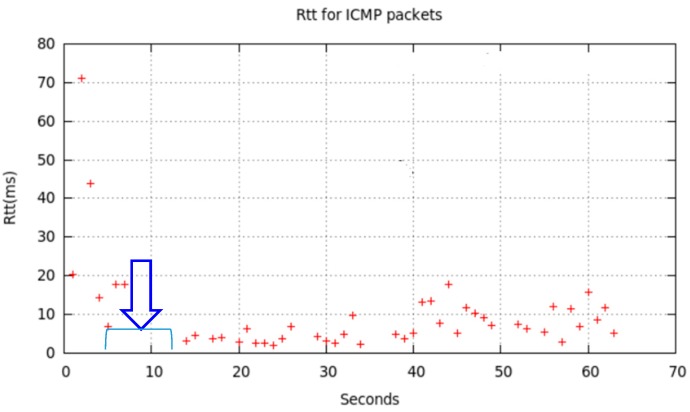
Effect on LFT packet sequence between node6 and node1.

**Figure 18:**
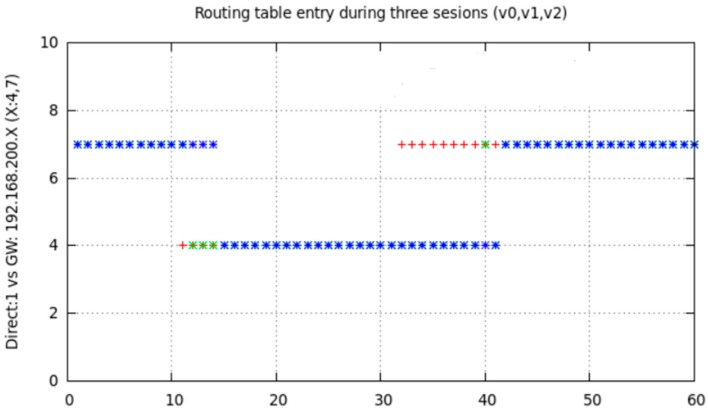
Chosen intermediate node for route from node6 to node1 in node6 for 3 HFT sessions of 60 s with node7 WiFi interface deactivation at *t* = 10 and *t_DOWN_* = 4 s.

We show in Figure 17 and Figure 18 the effect of changing from *MAP_ON_s* to *IFACE_DOWN_s* and return for non-sensitive LFT traffic and sensitive HFT traffic. Some LFT packets are lost due to node7 was deactivated at around *t* = 10 (blue arrow in Figure 17). We corroborated that from approximately *t* = 15 to *t* = 45 node6 used node4 as intermediate node, and later till *t* = 60 it used node7, as is shown in Figure 16. Figure 18 shows node6 routing table changes for three sessions, named v0 (red points), v1 (blue points) and v2 (green points), of HFT traffic. This communication consisted of the transmission from node6 of *temporal.tar* file (1,381,760 bytes) to node1. As in the previous tests, we deactivated node7 WiFi interface at instant *t* = 10, held it in this state for longer (specifically t_DOWN_ = 4 s), and later reactivated till end of transmission. The behaviour was similar in the three cases: route1 was initially used, it changed to route2, later returning to route1. The effect on reliable HFT traffic, under the user point of view, was hardly noticeable. Although the file was successfully received a communication disruption of 4 s occurred, due to OLSR could not configure immediately a new route after forcedly deactivating node7.

File transmission time-length in the three sessions was similar, about 60 s. Likewise, several TCP packets were lost during the table change early after *t* = 10. In all cases those packets were successfully retransmitted, ensuring a reliable communication despite node7 WiFi interface was deactivated while it was being used. In Table 7 we show the command line sequence for the file transmission. For all sessions (Figure 18) the route switched unpredictable from node7 to node4. This was unpredictable because it depended on whether (and when) the messages are received or the timers have expired. For example, in v0 session (red points), node6 selects again node7 at *t* = 32 unlike v1 session (blue point) at *t* = 42.

After these experimental tests we conclude: (a) intermediate nodes not participating in any route can deactivate its WiFi interface reducing its power consumption. (b) Intermediate nodes participating in a route also could deactivate its WiFi interface when their neighbours authorize it. This is specially useful for PWMNS.

#### 6.1.2. Interference Effects

In addition to the previous experiments we have evaluated the effect caused by traffic from external devices to the PWMNS over the same or overlapped channel. Specifically we studied the OLSR interchanged messages. Although shared use of WiFi spectrum is well documented [51], we will analyze the specific case of our test platform. We have configured four different cases:

(a) With very spurious signals on adjacent channels out of our control.
(b) With an AP on channel 11 (which is adjacent to the channel used on the test platform) and emitting only control frames (especially beacons) which we have called *Interfering_AP_1.*
(c) With an AP on channel 11 and another in channel 7 (same channel as our PWMNS configuration) which we have called *Interfering_AP_2*.
(d) *Interfering_AP_1* and *Interfering_AP_2*, but the latter transporting intensive user traffic (using *iperf* [52] application which we consider as interfering traffic for our network).

**Figure 19:**
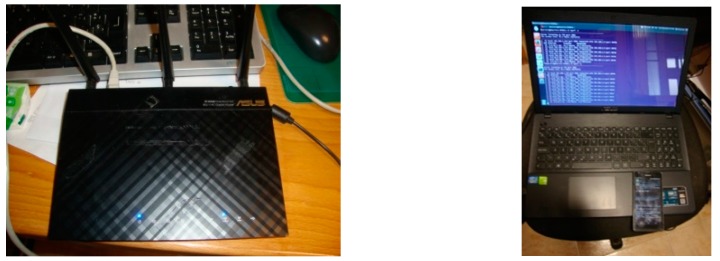
Main additional devices which generated interfering traffic.

Specifically *Interfering_AP_1* is an AP-router Belkin model F6D4630-4v1 operating on channel 11, *Interfering_AP_2* is an AP-router ASUS RT-AC66U Dual Band 3 × 3 802.11ac operating on channel 7. Additionally we used a laptop (ASUS model X552C operating under Ubuntu Linux) and a smartphone (SONY EXPERIA S35 operating under Android). These latter two end-devices were associated to *Interfering_AP_2* in infrastructure mode, and the laptop acting as an iperf server and the smartphone as an iperf client. Figure 19 shows an image of the latter three devices used in this experiment.

In case (a), our PWMNS is less interfered than the other cases. Once captured the OLSR messages detected by every node, we observed their high variability caused by the use of the same channel, hidden nodes and different conditions in the area close to every node.

**Figure 20:**
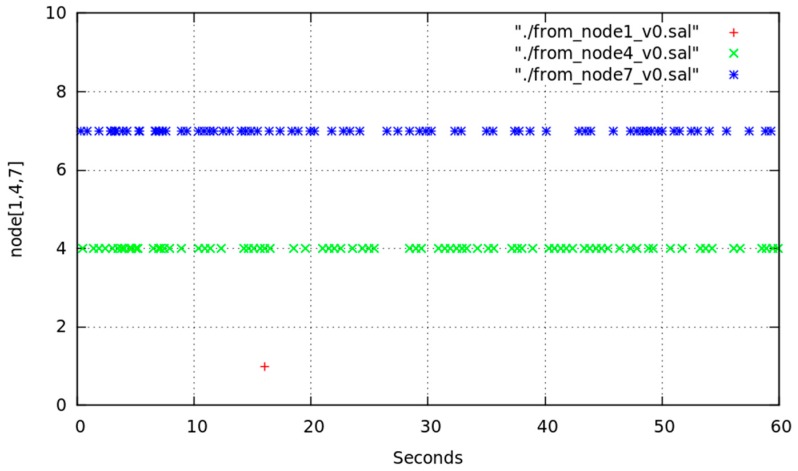
OLSR messages detected by node6.

We have highlighted in Figure 20 the origin (in the vertical axis) of the messages detected by node6 respect to the instant (horizontal axis) when they were detected. We have omitted the messages originated by node6. Let us notice the small number of messages from node1 while node4 and node7 transmitted a similar number of them.

We could better appreciate the effect on the interchanged messages between the different nodes when there existed other APs or devices emitting on adjacent channels and, specially, on the same channel 7. As we know shared-channel use caused restrictions when packets were emitted which, as consequence, derived in an emission reduction of OLSR messages as well as all other packets.

**Figure 21:**
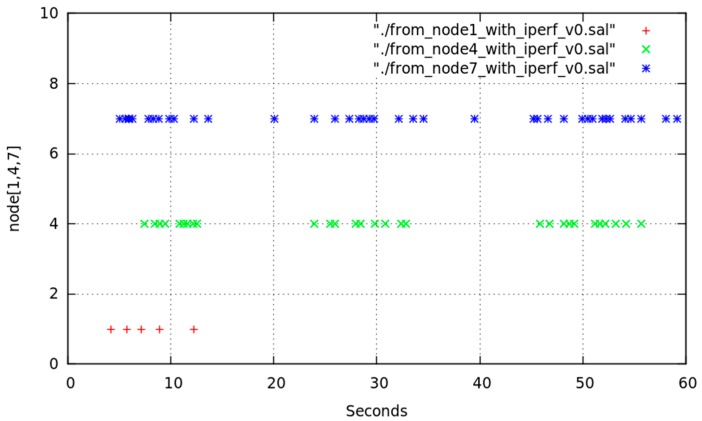
OLSR messages detected by node6 with channel 7 interferences at *t* = 10, *t* = 30 and *t* = 50.

This is shown in Figure 21 as a result of case (d). We have generated that interference traffic starting at instants *t* = 10, *t* = 30 and *t* = 50, and all of them have an approximately duration of 10 s (interference intervals: 10 ≤ *t* ≤ 20, 30 ≤ *t* ≤ 40 and 50 ≤ *t* ≤ 60). If we compare with Figure 20, we can clearly appreciate that, for the same 60 s session-interval, the number of messages detected by node6 coming from node1, node4 and node7 are fewer; especially those which should have been emitted at instants when *iperf* made an intensive use of the channel. If we analyse Figure 21 in depth, we corroborate the lack of OLSR messages, especially at instants soon after *t* = 10, *t* = 30 and *t* = 50.

The results for cases (b) and (c) (one single AP on channel 11 for the former; and one AP on channel 11 and another on channel 7 without user traffic) are not showed because they were very similar to case (a) with small variations on the number of detected messages.

Our proposal was based on the fact that, when several PWMNS nodes were interfered, OLSR message interchange with the rest of nodes (as well as nodes availability) were affected. The greater amount of external interferences were, the more severely the communication was affected. Thus participation of an interfered node in the PWMNS could become unnecessary (even it might negatively affect the communication), so that the deactivation of its wireless interface could be required.

**Figure 22:**
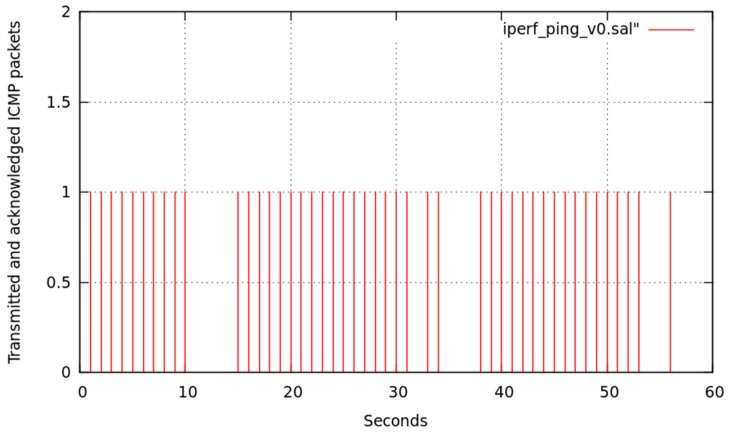
LFT messages confirmed by node6 from node1.

We show in Figure 22, the effects of this interfering traffic on channel 7 while LFT messages were sent from node6 to node1, coming from either node6 itself or any sensor (connected to node6) which generates such message pattern.

Figure 22 illustrates that, at instants 10 ≤ *t* ≤ 20, 30 ≤ *t* ≤ 40, and 50 ≤ *t* ≤ 60 (time intervals of iperf traffic generation), several LFT messages could not be emitted or their answers could not be received.

**Figure 23:**
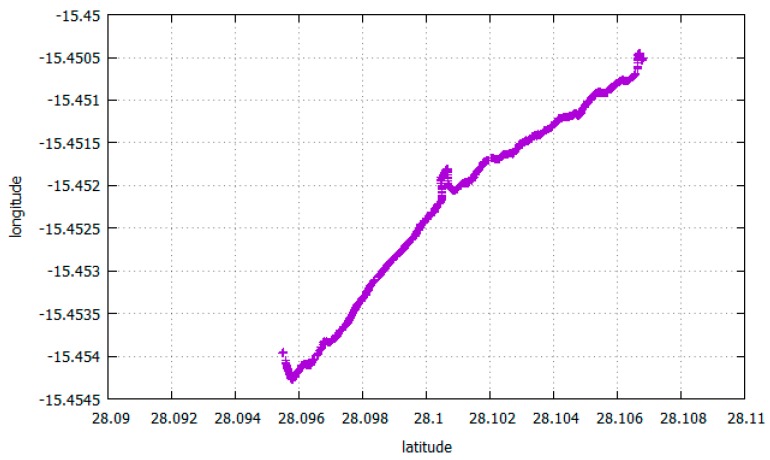
Location values with mobile phone outdoors.

**Figure 24:**
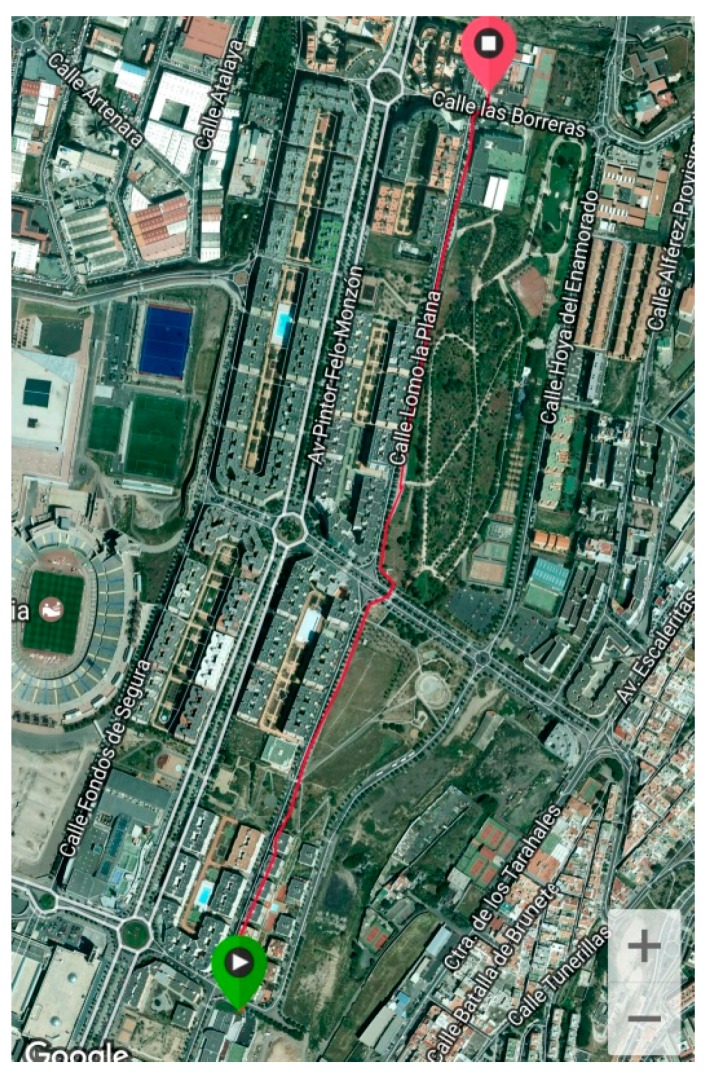
Satellite view of the route.

In order to analyse this LFT traffic we performed real measures of GPS with a Samsung Galaxy S7 Edge smartphone. Figure 23 shows raw location values (latitude and longitude) obtained outdoors. Figure 24 shows a satellite view of the 1.4 km long covered distance, where a pace of approximately 13 m 58 s per km was kept.

**Figure 25:**
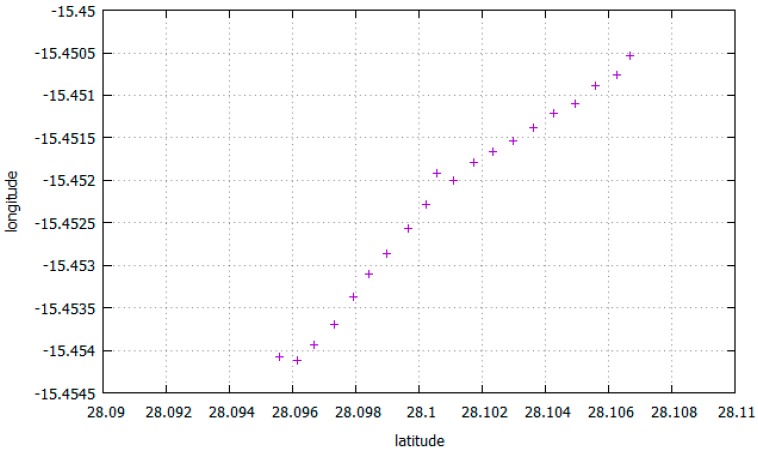
Average location values that are sent to the MAP every minute.

The location values can be sent as they are sensed to the corresponding MAP, or an app at the mobile phone can average a set of these values before doing the upload (e.g., the measures obtained every 10 m), or it can send them only every time there is a substantial location change, or at discretion of the sensing campaign. Figure 25 shows the average values every minute of the ones shown at Figure 23. The size of every sample is 24 bytes.

Its non-critical nature allows this smartphone sensing-data traffic to be easily processed and sent by our PWMNS. As the measures can be averaged, given its reduced variability (terminal positioning during a walker movement), those can be synchronized for a later sending, extending the interval between every pair of transmissions. Thus node inactivity periods are increased and a longer *IFACE_DOWN_s* state could be potentially ensured for any node. And hence power saving is increased and interference redress can be easily achieved.

### 6.2. Power Saving Calculation

**Figure 26:**
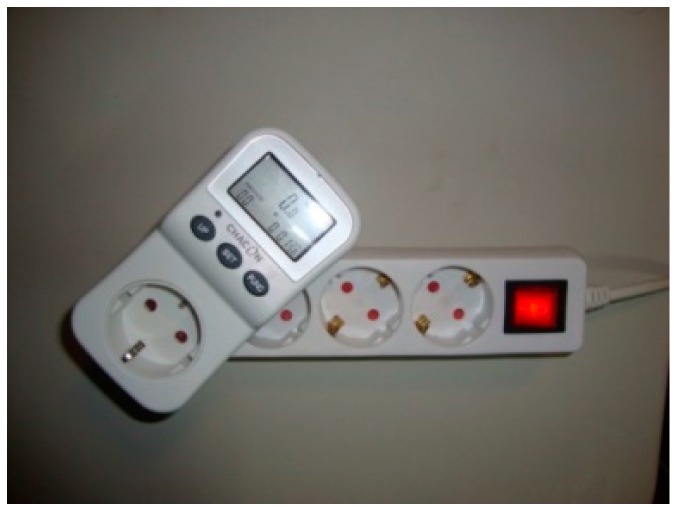
Wattmeter used in our experiment.

Figure 26 shows the device (Chacon Ecowatt wattmeter) we used for measuring the average-power consumed in every configuration. It also shows the null consumption without any charge.

We show in Table 8 the obtained measures of average-power consumed in the evaluated configurations, as indicated in Table 5.

If we calculate the average-power consumed by a node for a determined T interval, cycling its state from *MAP_ON_s* state to *IFACE_DOWN_s* state, and considering the calculation as indicated in Equation (2); we can estimate that consumption related to the inactivity experimented by that node in a specified time interval. Table 9 shows, for every configuration, its average-power consumed based on its consumption in its both possible states.

In order to evaluate the average power consumed for a time interval, considering a 60 s session (T = 60 s) and considering every node to be *MAP_ON_s* for a 75% of that time (45 s) and in *IFACE_DOWN_s* for a 25% (15 s), we have showed in Table 10 a comparative of average-power consumed for nodes used in our experiments. We can observe that, for the four configurations, the power saving was significant. It considers the average-power after both holding *MAP_ON_s* state and cyclic changing *IFACE_DOWN_s*-*MAP_ON_s* states.

In our test platform, as Section 6.1 describes, we program node7 for both a single change to *IFACE_DOWN_s* state at instant *t* = 10 and a dynamical change with different duty cycles for deactivation and activation. Specifically, assuming a repeating cycle of *MAP_ON_s* for 4 s and *IFACE_DOWN_s* for 2 s, node7 consumption drops to 149.4 W-min and global PWMNS consumption drops to 54.492 kWh.

## 7. Conclusions

In the last years WMNs have received close attention as an infrastructure for transporting traffic, being particularly noteworthy their role as sensing-data interconnection means throughout wide areas like smart cities. WMNs constitute a flexible solution which allows us to quickly deploy a backbone for sensing-data transport, across environments where alternative options (such as wired networks) are not appropriate. Furthermore, this deployment could be focused as a new concept, where sensors and end-users overlap with the backbone (WMN) thus shaping what we have called PWMNS, which allows a complete and portable solution for sensing data to be spread through the smart city. But this fast spread itself constitutes also an important problem, especially as regards average-power consumed by mesh nodes as well as harmonious coexistence with other wireless devices in the smart city.

In this paper we have proposed a mechanism for redressing the interferences caused, not only by external devices to our PWMNS, but also in the opposite direction. Thus we contribute towards the concept of Wireless-Friendliness, which should logically guide an environment where more and more wireless devices arise. On the other hand, our proposal also imposes a logical rationalizing of the average-power consumed by the PWMNS nodes, not only in order to extend their operational cycle in case of portable deployment, but also for reducing their carbon footprint. Hence, our PWMNS will intend any of its routes selected for connecting sensors with end-users to be established through nodes subjected to the lowest possible interference and which increase the power saving as much as feasible. Other works could be integrated in our mechanism for increasing the power saving. For this reason we showed with experimental results that our work increases power saving with respect to other related works.

We have sketched a concurrent set of routines which allows PWMNS nodes (along with a routing protocol as OLSR) to be coordinated for both redressing any experienced interference and increasing the power savings. Our simulation analysis has revealed that both targets may be simultaneously reachable. Although that simulation consisted of a straightforward 3 × 3 mesh, it helped in clarifying the meaning of our developed measures of interference redress $R^{Z}\left(t\right)$ and power saving $S^{Z}\left(t\right)$. Larger meshes were not possible to be tested due to the complexity increasing of finding all possible paths. Anyway, in a future work we intend to study the behaviour of our measures when randomly chosen paths are selected by OLSR under the *Network Simulator* (NS) environment.

We have used a test platform based on four RPis (routed by means of OLSR) for testing our new strategy of setting the WiFi interfaces of MAPs down. We did some measures indoors considering four different cases of interference patterns. For all cases we showed that MAPs can deactivate their WiFi interfaces, because OLSR always found a new route and our strategy forced them to receive authorization from their neighbours. We also did some GPS measures outdoors and showed that our PWMNS could deliver them efficiently. Finally we showed that our test platform could reach a power saving of approximately 10 Watt every minute, amounted to 600 Watt-hour for every node of this type. We plan to extend the test platform to show that with more potential routes to be found by OLSR, our strategy will work better redressing the adverse effects of interferences.

## Figures and Tables

**Table 1:** Different MAP states from the power consumption point of view.

State Name	MAP Wireless Interface Is	Full MAP System Is
*MAP_OFF_s*	down	off (it does consume no power)
*IFACE_DOWN_s*	down	on and its wireless interface is down
*MAP_ON_s*	up	on and its wireless interface is up

**Table 2:** Consumption measurements for the WRT160NL Router/AP.

State Name	Bandwidth	Power (Watts)
*MAP_ON_s* (b, g or n)	20 MHz	3.5
*MAP_ON_s* (n version only)	40 MHz	4.4
*IFACE_DOWN_s*	any	2.8

**Table 3:** List of messages the MAPs manage to set its wireless interface orderly down.

Message Name	Arguments	Direction	Description
*GO_IFACE_DOWN*	*t_DOWN_*, id_MAP	dMAP → neighbours	MAP is downable
ACK	*t_DOWN_*, id_MAP	neighbours → dMAP	Neighbour accept dMAP change to *IFACE_DOWN_s*
NACK	Motive (reason for refusal)	neighbours → dMAP	Neighbour refuse dMAP change to *IFACE_DOWN_s*
DOWN		dMAP → neighbours	End of negotiation

**Table 4:** Simulation results for a 3 × 3 PWMNS.

Route	R(t)	$\sum_{m=1}^{M}\sum_{n=1}^{N}\left\{D_{m,n}\text{​}\cdot \text{​}A_{m,n}\left(t\right)\right\}$	S(t)	$\sum_{m=1}^{M}\sum_{n=1}^{N}\left\{E_{m,n}\text{​}\cdot \text{​}A_{m,n}\left(t\right)\right\}$
1-4-5-8-9	1	0	0.3	1.5
1-4-7-8-9	1	0	0.3	1.6
1-2-5-8-9	0.8	0.2	0.7	0.9
1-2-5-4-7-8-9	0.8	0.2	0.1	1.8
1-4-5-6-9	0.4	0.5	0.6	1.1
1-4-7-8-5-6-9	0.4	0.5	0.1	1.8
1-2-5-6-9	0.1	0.7	1	0.5
1-2-3-6-9	0	0.8	1	0.5
1-2-3-6-5-8-9	0	0.8	0.6	1.1
1-4-5-2-3-6-9	0	0.8	0.5	1.3
1-2-3-6-5-4-7-8-9	0	0.8	0	2
1-4-7-8-5-2-3-6-9	0	0.8	0	2

**Table 5:** Configuration for the 4 RPi based PWMNS.

node4	RPi2: WiFi Linksys Cisco WUSB600N interface, Debian RPi kernel ver. 4.1.17-v7+
node1	RPi2: Alfa Chipset Realtek 8187L interface and 6 dBi antenna, Debian RPi kernel ver. 4.1.17-v7+
node6	RPi2+: Display LCD with WiFi Linksys Cisco WUSB600N interface, Debian RPi kernel ver. 4.1.17-v7+
node7	RPi3: internal WiFi interface Raspbian Jessie kernel ver. 4.4 (11 January 2017)

**Table 6:** Most habitual kernel IP routing table for node6.

Destination	Gateway	Genmask	Flags	Metric	Ref	Use IFace
169.254.0.0	0.0.0.0	255.255.0.0	U	303	0	0 wlan0
192.168.200.0	0.0.0.0	255.255.255.0	U	0	0	0 wlan0
192.168.200.1	192.168.200.7	255.255.255.255	UGH	2	0	0 wlan0
192.168.200.4	192.168.200.4	255.255.255.255	UGH	2	0	0 wlan0
192.168.200.7	192.168.200.7	255.255.255.255	UGH	2	0	0 wlan0

**Table 7:** Command line sequence for scp traffic.

pi@raspberrypi:~$ scp/home/pi/temporal.tar pi@192.168.200.1:/home/pi/temporal.tar			
pi@192.168.200.1’s password:			
temporal.tar	100%	13 MB 207.5 KB/s	01:05
pi@raspberrypi:~$ scp /home/pi/temporal.tar pi@192.168.200.1:/home/pi/temporal.tar			
pi@192.168.200.1’s password:			
temporal.tar	100%	13 MB 245.3 KB/s	00:55
pi@raspberrypi:~$ scp /home/pi/temporal.tar pi@192.168.200.1:/home/pi/temporal.tar			
pi@192.168.200.1’s password:			
temporal.tar	100%	13 MB 236.7 KB/s	00:57

**Table 8:** Average-power consumed in every configuration.

ModelIFaces	Without WiFi	Internal		WUSB600N		ALFA	
		Down	Up	Down	Up	Down	Up
RPI3 (node7)	-	2.0 W	2.7 W	-	-	-	-
RPI2 (node4, node1)	2 W	-	-	2.2 W	3.9 W	2.9 W	4.3 W
RPI2+LCD (node6)	3.4 W	-	-	3.2W (5.9 W) *	4.4 W (6.2 W) *	-	-

* includes monitor, keyboard and mouse extra average-power.

**Table 9:** Average-power consumed for an interval T = *t_UP_* + *t_DOWN_*.

node4	Average_power_T_ = 3.9 W × *t_UP_* + 2.2 W × *t_DOWN_*
node1	Average_power_T_ = 4.3 W × *t_UP_* + 2.9 W × *t_DOWN_*
node6	Average_power_T_ = 4.4 W × *t_UP_* + 3.2 W × *t_DOWN_*
node7	Average_power_T_ = 2.7 W × *t_UP_* + 2.0 W × *t_DOWN_*

**Table 10:** Average-power comparative for always *MAP_ON_s* or cyclic changing, for T = 60 s.

	Always *MAP_ON_s*	*IFACE_DOWN_s*-*MAP_ON_s*
node4	3.9 × 60 = 234 W-min	3.9 × 45 + 2.2 × 15 = 175.5 + 33 = 208.5 W-min
node1	4.3 × 60 = 258 W-min	4.3 × 45 + 2.9 × 15 = 193.5 + 43.5 = 237 W-min
node6	4.4 × 60 = 264 W-min	4.4 × 45 + 3.2 × 15 = 198 + 48 = 246 W-min
node7	2.7 × 60 = 162 W-min	2.7 × 45 + 2.0 × 15 = 121.2 + 30 = 151.2 W-min
Total (1 min)	918 W-min	842.7 W-min
Total (Watt-hour)	55.080 kWh	50.062 kWh

## References

[1] Akyildiz I.F., Xudong W. (2005). A survey on wireless mesh networks. IEEE Commun. Mag..

[2] Hossain E., Leung K.K. (2008). Wireless Mesh Networks.

[3] Lee M.J., Jianliang Z., Young-Bae K., Deepesh Man S. (2006). Emerging standards for wireless mesh technology. IEEE Wirel. Commun..

[4] Hiertz G., Denteneer D., Max S., Taori R., Cardona J., Berlemann L., Walke B. (2010). IEEE 802.11s: The WLAN mesh standard. IEEE Wirel. Commun..

[5] Bicket J., Aguayo D., Biswas S., Morris R. Architecture and Evaluation of an Unplanned 802.11b Mesh Network. Proceedings of the 11th Annual International Conference on Mobile Computing and Networking.

[6] Akyildiz I.F., Wang X., Wang W. (2005). Wireless mesh networks: A survey. Comput. Netw..

[7] He Y., Stojmenovic I., Liu Y., Gu Y. (2014). Smart city. Int. J. Distrib. Sens. Netw..

[8] Murty R.N., Mainland G., Rose I., Chowdhury A.R., Gosain A., Bers J., Welsh M. Citysense: An Urban-Scale Wireless Sensor Network and Testbed. Proceedings of the IEEE Conference on Technologies for Homeland Security.

[9] Bruno R., Conti M., Pinizzotto A. (2011). Routing internet traffic in heterogeneous mesh networks: Analysis and algorithms. Perform. Eval..

[10] Tang J., Xue G., Zhang W. Interference-Aware Topology Control and QoS Routing in Multi-Channel Wireless Mesh Networks. Proceedings of the 6th ACM International Symposium on Mobile Ad Hoc Networking and Computing.

[11] Raj M., Kant K., Das S.K. E-darwin: Energy Aware Disaster Recovery Network Using WiFi Tethering. Proceedings of the 23rd International Conference on Computer Communication and Networks.

[12] Lee K., Lee J., Yi Y., Rhee I., Chong S. (2013). Mobile data offloading: How much can WiFi deliver?. IEEE ACM Trans. Netw..

[13] Campista M.E.M., Esposito P.M., Moraes I.M., Costa L.H.M., Duarte O.C.M., Passos D.G., de Albuquerque C.V.N., Saade D.C.M., Rubinstein M.G. (2008). Routing metrics and protocols for wireless mesh networks. IEEE Netw..

[14] Johnson D., Hancke G. (2009). Comparison of two routing metrics in OLSR on a grid based mesh network. Ad Hoc Netw..

[15] Arce P., Guerri J.C., Pajares A., Lázaro O. (2008). Performance evaluation of video streaming over ad hoc networks using flat and hierarchical routing protocols. Mob. Netw. Appl..

[16] Sarmiento Á.S., La-Menza M., Macías E.M., Sunderam V.S. Automatic Resumption of Streaming Sessions over Wireless Communications Using Agents. Proceedings of the International MultiConference of Engineers and Computer Scientists.

[17] De la Oliva A., Banchs A., Serrano P. (2012). Throughput and energy-aware routing for 802.11 based mesh networks. Comput. Commun..

[18] Conti M., Giordano S. (2014). Mobile ad hoc networking: Milestones, challenges, and new research directions. IEEE Commun. Mag..

[19] Olwal T.O., Van Wyk B.J., Ntlatlapa N., Djouani K., Siarry P., Hamam Y. (2010). Dynamic power control for wireless backbone mesh networks: A survey. Netw. Protoc. Algorithms.

[20] Escolar S., Carretero J., Marinescu M.-C., Chessa S. (2014). Estimating energy savings in smart street lighting by using an adaptive control system. Int. J. Distrib. Sens. Netw..

[21] Cellucci L., Burattini C., Drakou D., Gugliermetti F., Bisegna F., Vollaro A., Salata F., Golasi I. (2015). Urban lighting project for a small town: Comparing citizens and authority benefits. Sustainability.

[22] Rodríguez-Molina J., Martínez J.-F., Castillejo P., de Diego R. (2013). Smarc: A proposal for a smart, semantic middleware architecture focused on smart city energy management. Int. J. Distrib. Sens. Netw..

[23] Li Q., Yang P. (2014). Ecots: Efficient and cooperative task sharing for large-scale smart city sensing application. Int. J. Distrib. Sens. Netw..

[24] Ha R.W., Ho P.H., Shen X. (2006). Optimal sleep scheduling with transmission range assignment in application-specific wireless sensor networks. Int. J. Distrib. Sens. Netw..

[25] Marrero D., Macías E., Suárez Á., Santana J.A., Mena V. (2016). A method for power saving in dense WiFi networks. Mob. Netw. Appl..

[26] Marrero D., Macías E., Suárez Á., Santana J.A., Mena V. (2016). Energy saving in smart city wireless backbone network for environment sensors. Mob. Netw. Appl..

[27] Khatoun R., Zeadally S. (2016). Smart cities: Concepts, architectures, research opportunities. Commun. ACM.

[28] Raspberri Pi Documentation. https://www.raspberrypi.org/documentation/.

[29] Van Der Schaar M., Sai Shankar N. (2005). Cross-layer wireless multimedia transmission: Challenges, principles, and new paradigms. IEEE Wirel. Commun..

[30] Macías E., Suárez A., Martín J., Sunderam V. (2007). Using olsr for streaming video in 802.11 ad hoc networks to save bandwidth. IAENG Int. J. Comput. Sci..

[31] Clausen T., Jacquet P. Optimized Link State Routing Protocol (OLSR). https://tools.ietf.org/html/rfc3626.

[32] Arce Vila P. (2014). Hierarchical Routing and Cross-Layer Mechanisms for Improving Video Streaming Quality of Service Over Mobile Wireless Ad Hoc Networks. Ph.D. Thesis.

[33] Kanchanasut K., Tunpan A., Awal M., Wongsaardsakul T., Das D., Tsuchimoto Y. (2007). Building a Long-Distance Multimedia Wireless Mesh Network for Collaborative Disaster Emergency Responses.

[34] Sun W., Wang H., Piao X., Qiu T. (2014). An opportunistic routing mechanism combined with long-term and short-term metrics for WMN. Sci. World J..

[35] Serrano S., Campobello G., Leonardi A., Palazzo S., Galluccio L. (2016). VoIP traffic in wireless mesh networks: A MOS-based routing scheme. Wirel. Commun. Mob. Comput..

[36] Li Z., Wang H., Dong C., Qian R. Urp: A Unified Routing Protocol for Heterogeneous Wireless Mesh Networks. Proceedings of the IEEE Wireless Communications and Networking Conference.

[37] Li Z., Wang H., Dong C., Wu F., Yu W. (2016). Unified routing protocol based on passive bandwidth measurement in heterogeneous WMNs. Wirel. Commun. Mob. Comput..

[38] Rigelsford J., Ford K., Yu T., Lai Z., Valtr P., Weng J., Wang Y., Vallecchi A., Altan H., Song H. Wireless Friendly and Energy Efficient Buildings (WiFEEB). Proceedings of the Progress in Electromagnetics Research Symposium Abstracts.

[39] Alotaibi E., Mukherjee B. (2012). A survey on routing algorithms for wireless ad-hoc and mesh networks. Comput. Netw..

[40] Vijayakumar K., Ganeshkumar P., Anandaraj M. (2012). Review on routing algorithms in wireless mesh networks. Int. J. Comput. Sci. Telecommun..

[41] Ahmeda S.S., Farhan R.K. Routing protocols for wireless mesh networks. Proceedings of the 1st International Congress on Computer, Electronics, Electrical, and Communication Engineering.

[42] Marrero D., Macías E.M., Suárez A. (2008). An admission control and traffic regulation mechanism for infrastructure WiFi networks. IAENG Int. J. Comput. Sci..

[43] Dely P., D’Andreagiovanni F., Kassler A. (2015). Fair optimization of mesh-connected WLAN hotspots. Wirel. Commun. Mob. Comput..

[44] Nandiraju N., Nandiraju D., Agrawal D. Multipath Routing in Wireless Mesh Networks. Proceedings of the IEEE International Conference on Mobile Adhoc and Sensor Systems.

[45] Wireless-g Broadband Router. http://www.linksys.com/us/support-product?pid=01t80000003KXPxAAO.

[46] Unleash Your Router. http://www.dd-wrt.com/site/index.

[47] Calculator for Consumption in Standby. http://www.ocu.org/vivienda-y-energia/nc/calculadora/consumo-en-stand-by.

[48] Linksys Official Support—Linksys WRT160NL Wireless-N Broadband Router with Storage Link. http://www.linksys.com/us/support-product?pid=01t80000003K7eJAAS.

[49] Marrero Marrero D. (2016). Characterizing and Modelling Wireless Network Performance for Applications with Quality of Service. Ph.D. Thesis.

[50] Santana J.A., Macías E., Suárez Á., Marrero D., Mena V. (2016). Adaptive estimation of WiFi RSSI and its impact over advanced wireless services. Mob. Netw. Appl..

[51] Lakshminarayanan K., Seshan S., Steenkiste P. Understanding 802.11 performance in heterogeneous environments. Proceedings of the 2nd ACM SIGCOMM Workshop on Home networks.

[52] iPerf—The Ultimate Speed Test Tool for TCP, UDP and SCTP. https://iperf.fr/.

